# The partnership of patient advocacy groups and clinical investigators in the rare diseases clinical research network

**DOI:** 10.1186/s13023-016-0445-8

**Published:** 2016-05-18

**Authors:** Peter A. Merkel, Michele Manion, Rashmi Gopal-Srivastava, Stephen Groft, H. A. Jinnah, David Robertson, Jeffrey P. Krischer, for the Rare Diseases Clinical Research Network

**Affiliations:** Division of Rheumatology and Department of Biostatistics and Epidemiology, University of Pennsylvania, Philadelphia, PA USA; PCD Foundation, Minneapolis, MN USA; Office of Rare Diseases Research (ORDR), National Center for Advancing Translational Sciences (NCATS), National Institutes of Health, Bethesda, MD USA; National Center for Advancing Translational Sciences (NCATS), National Institutes of Health, Bethesda, MD USA; Departments of Neurology, Human Genetics and Pediatrics, Emory University School of Medicine, Atlanta, GA USA; Departments of Medicine, Pharmacology, and Neurology, Clinical Research Center, Vanderbilt University, Nashville, TN USA; Health Informatics Institute and Department of Pediatrics, University of South Florida, 3650 Spectrum Boulevard, Tampa, FL 33612 USA

**Keywords:** Rare diseases, Patient engagement, Network

## Abstract

**Background:**

Among the unique features of the Rare Diseases Clinical Research Network (RDCRN) Program is the requirement for each Consortium to include patient advocacy groups (PAGs) as research partners. This development has transformed the work of the RDCRN and is a model for collaborative research. This article outlines the roles patients and PAGs play in the RDCRN and reports on the PAGs’ impact on the Network’s success.

**Methods:**

Principal Investigators from the 17 RDCRN Consortia and 28 representatives from 76 PAGs affiliated with these Consortia were contacted by email to provide feedback via an online RDCRN survey. Impact was measured in the key areas of 1) Research logistics; 2) Outreach and communication; and 3) Funding and in-kind support. Rating choices were: 1-very negative, 2-somewhat negative, 3-no impact, 4-somewhat positive, and 5-very positive.

**Results:**

Twenty-seven of the PAGs (96 %) disseminate information about the RDCRN within the patient community. The Consortium Principal Investigators also reported high levels of PAG involvement. Sixteen (94 %) Consortium Principal Investigators and 25 PAGs (89 %) reported PAGs participation in protocol review, study design, Consortium conference calls, attending Consortium meetings, or helping with patient recruitment.

**Conclusions:**

PAGs are actively involved in shaping Consortia’s research agendas, help ensure the feasibility and success of research protocols by assisting with study design and patient recruitment, and support training programs. This extensive PAG-Investigator partnership in the RDCRN has had a strongly positive impact on the success of the Network.

## Background

A “rare disease” is defined by the Amendment to the Orphan Drug Act of 1983 as a condition affecting fewer than 200,000 Americans or a disease with a greater prevalence but for which no reasonable expectation exists that the costs of developing or distributing a drug can be recovered from the sale of the drug in the United States [1]. Approximately 25 million people in the United States are affected by one or more of an estimated 7000 rare diseases or conditions. These diseases often lead to significant morbidity and mortality.

Research into rare diseases encounters unique challenges to the scientific community, the biopharmaceutical and medical device and diagnostics industries, academic and public sector researchers, government funding agencies, private foundations, regulatory agencies, and patient advocacy groups. These challenges include difficulties in establishing diagnoses, difficulty in recruiting subjects into clinical studies due to small patient populations distributed over wide geographic areas, few expert centers for diagnosis, management, and research, and a scarcity of investigators focused on these rare diseases. These issues continue to be addressed with steady advances being made in the approach to studying rare diseases and, in parallel, substantial interest by the biopharmaceutical industry in developing products for the treatment of rare diseases.

To respond to the challenges of research on rare diseases the NIH Office of Rare Diseases Research (ORDR), now located in the National Center for Advancing Translational Sciences (NCATS), established the Rare Diseases Clinical Research Network (RDCRN) in 2003, in collaboration with six other Institutes and Centers (ICs) and funded 10 Rare Diseases Clinical Research Consortia (RDCRC) and a single Data Management and Coordinating Center (DMCC) for the whole Network [2]. The RDCRN was expanded in 2014, in collaboration with ten NIH ICs and currently consists of 22 research consortia and a central DMCC. Each Consortium is required to focus on a group of at least three related disorders, include multiple investigators at multiple sites, and collaborate with patient advocacy groups (PAGs). The RDCRN is unique in its approach to addressing rare diseases as a group, including promoting collaboration with PAGs, and is the first program that promised to create a collaborative and coordinated network of investigators and patient groups to support research into rare diseases.

The RDCRN has proven to be an extremely effective research model to maximize research investigator participation, initiating clinical trials, facilitating patient recruitment with established research partnerships with PAGs at multiple research sites around the world, and enabling pharmaceutical industry and government sponsored clinical research studies to proceed with a supportive infrastructure to complete the studies in a timely fashion [3].

Among the unique features of the RDCRN is the requirement for each Consortium to include PAGs as research partners. This mandate has led to the development of a vibrant culture within the Network in which the roles of patients has grown steadily to the current state in which patients are highly engaged in every aspect of the work of the Consortia. This article outlines the roles patients and PAGs play in the RDCRN and reports on data collected to evaluate these roles and their impact on the Network’s success.

### Background, goals, and organization of the RDCRN

The purpose of the RDCRN is to facilitate multi-site clinical research in rare diseases through support for 1) collaborative clinical research in rare diseases, including longitudinal studies of individuals with rare diseases, clinical studies and/or phase I, II, and II/III trials; 2) training of clinical investigators in rare diseases research; 3) pilot and demonstration projects; 4) a test bed for distributed clinical data management that incorporates novel approaches and technologies for data management, data mining, and data sharing across rare diseases, data types, and platforms; 5) collaboration with PAGs as research partners; and 6) access to information related to rare diseases for basic and clinical researchers, academic and practicing physicians, patients, and the lay public. The RDCRN, with the input from PAGs associated with Consortia, has been successful in providing website resource for education and research in rare diseases for health care providers, patients and their families, the biopharmaceutical and medical device industry, and research investigators [3]. Included in the RDCRN is an active Patient Contact Registry to facilitate the recruitment of patients and communications between investigators and patients and their families [4–6]. PAGs have contributed more than 40 % to the total numbers enrolled.

More than 70 PAGs are active participants in the RDCRN. Through the direct engagement of patients, their families and friends, PAGs led to acceptance of these individuals and organizations as research partners and the establishment of the Coalition of Patient Advocacy Groups (CPAG) for the RDCRN. CPAG represents the perspective and interests of all PAGs associated with the RDCRN and includes representatives from every PAG in the RDCRN, has its own governing structure and leadership, and is a major component of the RDCRN with voting privileges on the RDCRN Steering Committee. The RDCRN-CPAG meets annually, at the same time as the RDCRN Steering Committee, and has standing conference calls throughout the year.

### Value of patient advocacy groups as research partners

PAGs within the RDCRN are involved as research partners, including helping to recruit for clinical studies, encouraging participation in natural history studies, identifying cohorts of patients with a range of phenotypic expression, and educating patients, public, media and health care providers. Several PAGs provide financial support for research and training programs of RDCRC and patient registries. In addition, PAGs identify research efforts and translate research results to communities, organize and fund research based scientific conferences and meetings for patients/families/caregivers. A few PAGs also provide financial support for patients to travel to clinics to facilitate access to investigators and studies.

Each Consortium in the RDCRN includes relevant PAGs in the Consortium membership and activities and the direct involvement of PAGs in RDCRN operations, activities, and strategy is a major feature of this network.

## Methods

Principal Investigators (PIs) from the 17 RDCRN Consortia established at time of this study and representatives from PAGs affiliated with these Consortia were contacted by email and asked to provide feedback via an online RDCRN survey. Two complementary survey tools were developed and implemented in parallel. The first survey focused on investigators’ perceptions of the impact of Consortium/PAG collaboration; a second survey, with identical or substantially similar questions, focused on PAGs’ perceptions of their impact on the RDCRN. The surveys contained descriptors of the most commonly reported types of interactions.

Both versions of the survey consisted of seven questions. Four multi-part rating scale questions were designed to elicit the perceptions of impact of the RDCRN collaborative research model on achieving research goals from the perspective of the Consortium PI compared with the perceptions of the PAGs. Impact was measured in the key areas of 1) Research logistics, including study design, patient recruitment and administrative support; 2) Outreach and communication, including website design, patient educational meetings and development of educational materials; 3) Funding support for RDCRC activities; and 4) Other non-monetary forms of support. Rating choices were scored as follows: 1-very negative, 2-somewhat negative, 3-no impact, 4-somewhat positive, and 5-very positive. There was also an option for “not applicable.” Responses in each category were tabulated for an average score. Average scores for each category were compared between the Consortium PI responses and the PAG responses.

Additionally, two questions asked each respondent to identify the top three benefits and top three challenges of working collaboratively within the RDCRN model from a prepopulated list of 17 topics selected through consensus by representatives of the RDCRN Strategic Planning Committee, the sub-committee of the RDCRN Steering Committee that oversaw the survey project. The final question utilized a free-text answer and provided an opportunity for respondents to include additional commentary regarding experiences with the RDCRN collaborative research model.

### Data analysis

Responses were matched by Consortium and then anonymized so as to mask respondents and their assessments. Since there were multiple PAG respondents for several Consortia, the median response was used to pair with the corresponding Consortium response. Analyses were descriptive and both means and medians calculated. As the responses were on a Likert-type scale ranging from 1 to 5, medians are generally considered the best measure of central tendency, but means are also presented. On an interval scale, medians are also less affected by outliers and the difference can be interpreted as to the extent there are wide variations in response. As these are purely descriptive data with a high degree of concordance, no attempt was made to test whether or not the row and column marginal frequencies are equal.

## Results

All 17 Consortia PIs and 28 of 76 PAGs associated with the RDCRN completed the survey, with at least one PAG representing each Consortium (range 1–3). Except for one, the responding PAGs were established before 2010, with 13 established before 1990. There was a large range in the number of patient/families served by these PAGs and the reported numbers were equally split between less < 1000, 3000–5000, and ≥10,000. 50 % of responding PAGs reported a financial capacity of more than $1 million, and all but 6 reported having their own research funding program. The Consortia PIs reported that 82-100 % of their affiliated PAGs (not just of those who responded to the survey) collaborated on the specific items listed while 48-96 % of the responding PAGs reported collaboration along these same dimensions (Table 1).

**Table 1:** RDCRN consortium-patient advocacy group partnership survey response

Survey question: what impact has your (associated) PAG(s) had with your RDCRN consortium activities in each of the following areas?	Consortium PIs (*N* = 17)			PAGs (*N* = 28)		
	Engage in activity (%)	Mean	Median	Engage in activity (%)	Mean	Median
Research						
Review protocols and provide substantive input on study design	14 (82)	4.2	5	21 (75)	4.5	5
Review study forms and other study related documents	16 (94)	4.3	4	19 (68)	4.6	5
Participate in Consortium conference calls	16 (94)	4.6	5	25 (89)	4.7	5
Attend Consortium investigator meetings	16 (94)	4.8	5	21 (75)	4.8	5
Help with patient recruitment for RDCRN studies	16 (94)	4.7	5	24 (86)	4.5	5
Provide logistical support for Consortium meetings, calls, etc.	14 (82)	4.2	4.5	18 (64)	4.3	5
Provide administrative support to Consortium	14 (82)	3.8	3.5	14 (50)	4.3	4.5
Communication/Outreach						
Contribute to Consortium website design and content	14 (82)	4.5	5	22 (79)	3.9	4
Include Consortium activities, updates or relevant sessions at PAG meetings	17 (100)	4.7	5	24 (86)	4.6	5
Communicate Consortium activities within the patient community through website, newsletters, etc.	17 (100)	4.8	5	27 (96)	4.7	5
Provide educational materials related to Consortium activities for patient community	15 (88)	4.9	5	23 (82)	4.6	5
Funding						
Provide direct funding to the Consortium	13 (76)	4.5	5	16 (57)	4.3	5
Provide funding support for Consortium meetings/activities	14 (82)	4.4	5	18 (64)	4.1	4
Provide partial or full funding for Consortium trainees	12 (71)	4.2	4.5	13 (46)	4.2	4
Provide in-kind support (not necessarily funding) for Consortium activities (e.g., mailings, office staff, other)	14 (82)	4.4	4	17 (61)	4.4	5

*RDCRN* Rare Diseases Clinical Research Network, *PIs* principal investigators, *PAG* patient advocacy group

Scale: 1 = very negative, 2 = somewhat negative, 3 = no impact, 4 = somewhat positive, 5 = very positive. *N* total number of Consortium PIs or PAGs that participated in the survey

### Interactions

The most commonly reported interactions between Consortia and PAGs were those that involved communication and outreach to the patient community. Nearly all PAGs (27/28, 96 %) disseminate information about Consortium activities within the patient community via their PAG websites, newsletters, and other forms of communication. All PIs and 86 % of PAGs include updates for their associated Consortium during their PAG meetings. Most PIs (88 %) and PAGs (82 %) also provide patients with educational materials related to Consortium activities. The areas in which the PAGs reported less collaboration included providing partial or full funding of Consortium trainees (46 %), administrative support to the Consortium (50 %), direct funding to the Consortium (57 %), logistical support to the Consortium for meetings (64 %), and review of study forms and other study related documents (68 %).

The Consortium PIs also reported high levels of PAG involvement in Consortium research activities. Sixteen of the 17 (94 %) Consortium PIs and 25 of the 28 (89 %) responding PAGs reported PAG participation in protocol review, study design, Consortium conference calls, attending Consortium meetings, or helping with patient recruitment. These data suggest active PAG involvement in shaping the Consortia’s research agenda and contributing to ensuring the feasibility and success of research protocols by assisting with patient recruitment. Additionally, 82 % of PIs and 64 % of PAGs indicated that the PAGs provided logistical or administrative support to their Consortium.

All but one PAG indicated that they played an active role in communicating Consortium activities to their membership through social media, newsletters and other means. These are the primary means by which PAGs mobilized their membership to enhance recruitment efforts. A substantial percentage (82-86 %) of PAGs provided time at their meetings for presentation and discussion of Consortium activities and dissemination of educational materials developed by their affiliated Consortium. A similar percentage (79 %) of PAGs also contributed to the Consortium website development. In the RDCRN, each Consortium maintains a public-facing web presence to communicate to the larger community of investigators, care givers, and patients about the Consortium’s specific collection of rare diseases.

Thirteen (76 %) PIs indicated that their Consortia receive direct funding from one or more of their associated PAG(s). In all but one instance, this support included partial or full funding of RDCRN trainees (including travel to meetings). Nine of the 13 (69 %) Consortium PIs reported that they have received a total of at least $100,000 from their associated PAGs. One PAG has provided $2.25 million in direct financial support for a Consortium’s activities. Two (12 %) Consortium PIs reported the level of support to be between $10,000 and $49,000, and 2 did not indicate a level of funding. Eighteen of the 28 (64 %) PAGs responding indicated they provide funding support for Consortium meetings/activities. In addition to direct funding, 17 of the 28 (61 %) PAGs provide in-kind support for Consortium activities, such as mailings or office staff. One of the four Consortia that did not receive direct funding reported that they received in-kind support.

### Impact

When PAG responses were compared to those from the PI of their affiliated consortium, there was a high degree of concordance (Table 2). Averaging over all questions, there were no Consortia responses less than “somewhat positive” (a score of 4.0) and 14 of 17 ranked the interaction as “very positive” (a score of 5.0). For the Consortium PIs, the ratings ranged from a mean of 3.8 (“provide administrative support to a Consortium,” the only item in which the mean was less than 4.0) to 4.9 (“provide educational materials related to Consortium activities for patient community”). Similarly, only 1 of the 17 Consortia received an average score across all responses of 3 (“no impact”), the remainder were either “very positive” or “somewhat positive” (9 and 7, respectively). The item that received the lowest PAG rating, with a mean score of 3.9, was “contribute to Consortium website design and content.” That was the only item in which the mean was less than 4.0) and the item with the highest mean rating for impact was 4.8 (“attend Consortium investigator meetings”).

**Table 2:** Concordance between consortium principal investigators and corresponding patient advocacy group responses

	Average of corresponding PAG responses				
Average PI response	Very negative	Somewhat negative	No impact	Somewhat positive	Very positive
very negative	0	0	0	0	0
somewhat negative	0	0	0	0	0
no impact	0	0	0	0	0
somewhat positive	0	0	1	3	2
very positive	0	0	0	4	7

*PIs* principal investigator, *PAG* patient advocacy group

When asked to rank the top three benefits accrued to the Consortium through their interactions with PAGs (Table 3), the most frequently cited benefits were help with patient recruitment for RDCRN studies (11 of 15 respondents, 73 %), communication of Consortium activities within the patient community (6 of 15 respondents, 40 %), and providing direct funding to the Consortium (4 of 15 respondents, 27 %). The top three benefits for the PAGs were participation in Consortium conference calls (15 of 28 respondents, 54 %), inclusion in Consortium activities (14 of 28, 50 % or respondents), and help with patient recruitment for RDCRN studies (13 of 28 respondents, 46 %). The other benefits reported were distributed widely across respondents with 3 or fewer Consortia and 6 or fewer PAG respondents emphasizing their benefit.

**Table 3:** Top benefits of consortium-patient advocacy group interactions

	Principal investigators		Patient advocacy groups	
Benefit	No. of PIs selecting	% of 15 investigators answering question	No. of PAGs selecting	% of 28 PAGs answering question
Attend Consortium investigator meetings	3	20 %	14	50 %
Communicate Consortium activities within the patient community	6	40 %	6	21 %
Contribute to Consortium website design and content	2	13 %	1	4 %
Help with patient recruitment for RDCRN studies	11	73 %	13	46 %
Include Consortium activities, updates or relevant sessions at PAG meetings	1	7 %		
Include Consortium activities, updates or relevant sessions at PAG meetings			6	21 %
Other communication activities			3	11 %
Other research activities	1	7 %	4	14 %
Participate in Consortium conference calls	3	20 %	15	54 %
Provide administrative support to Consortium	1	7 %		
Provide direct funding to the consortium	4	27 %	3	11 %
Provide educational materials related to Consortium activities for patient community	2	13 %		
Provide funding support for Consortium meetings/activities	2	13 %		
Provide logistical support for Consortium meetings, calls, etc.	3	20 %	1	4 %
Provide partial or full funding for Consortium trainees	3	20 %	1	4 %
Received funding or other support from Consortium			2	7 %
Review protocols and provide substantive input on study design	2	13 %	2	7 %
Review study forms and other related documents	1	7 %	3	11 %

*PIs* principal investigators, *PAG* patient advocacy group

All respondents were also asked to provide feedback on their top 3 challenges in working collaboratively (Table 4). These choices were drawn from the same list as the benefits, and reflecting those results, the top challenge for Consortia PIs was funding (4 of 15 responses) and, for PAGs, desire for input into protocol development (6 of 26 responses).

**Table 4:** Top challenges of consortium-patient advocacy group interactions

	Principal investigators		Patient advocacy groups	
Benefit	No. of PIs selecting	% of 15 investigators answering question	No. of PAGs selecting	% of 26 PAGs answering question
Attend Consortium investigator meetings	1	7 %	3	12 %
Communicate Consortium activities within the patient community			2	8 %
Contribute to Consortium website design and content	1	7 %	1	4 %
Help with patient recruitment for RDCRN studies	1	7 %	4	15 %
Include Consortium activities, updates, or relevant sessions at PAG meetings			1	4 %
Other communication activities			4	15 %
Other research activities	1	7 %		
Participate in Consortium conference calls	2	13 %	3	12 %
Provide administrative support to Consortium	1	7 %		
Provide direct funding to the Consortium	4	27 %	1	4 %
Provide funding or other support to PAGs	1	7 %		
Provide funding support for Consortium meetings/activities	1	7 %		
Provide partial or full funding for Consortium trainees	2	13 %	1	4 %
Received funding or other support from Consortium			2	8 %
Review protocols and provide substantive input on study design	2	13 %	6	23 %
Review study forms and other related documents	1	7 %	4	15 %

*PIs* principal investigators, *PAG* patient advocacy group

Table 5 contains the individually-reported comments from the Consortium PIs and the PAGs to give a broader flavor of the nature of the interactions.

**Table 5:** Individual free text comments from RDCRN consortium principal investigators and patient advocacy groups

Patient advocacy group
Development and growth of the Consortium-sponsored symposia has had a tremendously positive effect on the rare disease community.
Assisting in recruitment through specific protocols and through conferences has been very rewarding. Additionally data derived on and subsequently published has made an immediate impact to the patient and medical community. This has been made possible through RDCRN. Working with other investigators and PAGs has also provided opportunities to learn from each other through professional interaction.
A big challenge was understanding the limits of the contact registry. [The PAG is] responsible for getting >91 % of patients who are registered. Yet, we could not access those patients nor did the patients receive significant benefit
I am very happy to be the PAG representative. It would be wonderful if someone took notes on conference calls for those who cannot make them.
During the first couple of years the PAGs had a monthly conference call with the project manager to discuss what was going on. Would be good to reinstate these calls - maybe on a quarterly basis to keep PAGs up-to-date.
Expanding our relationship with our PIs through Consortium activities has been one of the most fulfilling parts of our involvement in the RDCRN. The Consortium has been a wonderful platform to expand our outreach & growth to the affected community. It has been wonderful to work with investigators and PAGs from other Consortia and to learn from their experiences. We are forever grateful to the RDCRN and hope to continue this partnership for years to come.
In the first few years of our Consortium it was an uphill rocky climb to get researchers to accept and trust our PAG input related to how best to engage and recruit participants and to enroll participating research sites. With continued communication and low turnout becoming evident the tide has now turned for the better and we are seeing our input now valued and increased patient participation.
Our PAG became very involved with the RDCRN in about 2005, and involvement has been steady and positive since that time, but ebbs and flows with renewals and travel site scheduling. Involvement in the study design is highest and most collaborative right now in 2013 with our PAG interest in 2014 renewal. RDCRN has been very responsive to our PAG on any data questions and publishing on issues important to our community that can be revealed by the database.
Our PAG is pleased with the positive results of our collaboration.
Our Consortium is quite different from most others in that there is not a clinical trial associated with it (yet). The focus is on basic and translational research and the PAG-researcher relationship has been very constructive in that space.
The [Consortium] PAGs are still mostly arm's length from operations. Leadership wants to be open but on a practical basis operates in a fairly closed circle.
The cooperation and communication between the PAGs and the PIs has been wonderful. It has strengthened the community as we feel we are all working together for a common goal. The researchers respect and embrace the role the PAGs play to help support the Consortium and together we are moving research forward impacting the lives of patients. It has also allowed for smaller PAGs to make an impact on research by supporting programs like Travel Scholarships helping with recruitment and promoting the work of the [Consortium].
Time is a challenge - we would like to participate more. We include Consortium activities in our newsletter.
Wonderful collaborative relationship and feel like we have been accepted as equal partners.
Principal investigator
Our relationship with our associated PAGs is excellent and our PAGs are an essential component to the success of our Consortium.
Overall the Consortium has benefited significantly from the support of PAG groups affiliated with it.
Our PAGs have been very supportive in communicating our study and research activities to the patient community, and providing in-kind support by sending out letters describing our studies or printing educational materials, etc. They have provided some trainee support in the form of travel scholarships to attend respective scientific meetings hosted by the PAG.
Support from our PAGS has allowed us to add multiple sites to our RDCRC. Without their support we would not be able to accomplish much of what we have done over the past 4 years
The PAG has been involved in all aspects of the Consortium activities and all interactions have been only positive
The PAGs are an enormous asset to the Consortia and they are becoming a greater asset as time goes forward.
The PAGs try very hard to help and they do help meaningfully in some ways. They are very busy have day jobs and little resources. Also they are lay people do not express interest in reviewing study documents.
The regular intellectually invested and committed involvement of our PAG is the key benefit… it really helps to have constant feedback and input. It would be a bonus if they could be able to provide some funding but that is a secondary issue for us.
The partnership of our PAGs and the Consortium has been very effective toward achieving our common mission of advancing understanding of these diseases developing more effective treatments and assuring that all patients have access to current correct information. Much more is achieved by this partnership than would be possible by either of us working alone.

## Discussion

This study describes the successful approaches and common challenges in directly engaging patients and PAGs with investigators in the creation, growth, and productivity of multicenter research groups involved in clinical research in rare diseases. The ten-year experience of the RDCRN has been one of great success and scientific productivity. The RDCRN investigators, affiliated PAGs and patient leaders, ORDR-NCATS and collaborating Institutes’ program staff at NIH, and other key stakeholders all agree that the substantial partnership and involvement of patients, from the start, has been a major factor in the success of the Network and helped the Consortia conduct important research in a large of number of rare diseases. The investigator-patient partnership has contributed to the development of the Network on multiple levels and patients and PAGs have been involved in research in the RDCRN in a wide variety of ways.

The results of this study describe numerous ways in which PAGs and RDCRN investigators work together towards common goals for many different types of studies. This degree of involvement by PAGs is not typical of most NIH-funded research. There was strong agreement among both PAG representatives and RDCRN investigators that these collaborations usually had a positive impact.

One of the major challenges for any clinical study is recruitment of appropriate participants in a timely manner. Through their existing web sites and often regular patient support group meetings, the PAGs positively influence recruitment for clinical studies by educating potential participants about the value of clinical studies in general, advertising specific studies directly, answering questions about specific studies, and providing feedback on outcomes in some cases. In view of these factors, it is not surprising that communication and outreach were ranked among the most impactful aspects of interaction by both PAGs and RDCRN investigators.

In many cases, PAGs have a direct influence on the types of scientific projects that are initiated by providing direct funding for specific projects or specific investigators. The investigators, in turn, sometimes provide advice on independent scientific projects that the PAGs may be considering through service on their scientific advisory boards. Additionally, many PAGs recognize the importance of attracting a new generation of investigators and they often provide financial support for trainee-led research projects or trainee attendance at Consortium or other relevant meetings. All of these activities were also rated as having important impact by both PAGs and RDCRN investigators.

The RDCRN, with the input from PAGs associated with Consortia, has been successful in providing website resource for education and research in rare diseases for health care providers, patients and their families, the biopharmaceutical and medical device industry, and the research investigators. Many PAGs have at least some educational material on their web sites, brochures, or newsletters, and the RDCRN Consortia have similar educational material on their web sites. A close collaboration between PAGs and investigators can be useful in confirming the accuracy of the information provided and ensuring the language used is written at a level and in a style accessible to most patients and their families.

An important and unique component of the RDCRN is an active Patient Contact Registry to facilitate the recruitment of patients and communications between investigators and patients and their families. PAGs are the single largest source of referrals to the Patient Contact Registry [7, 8].

The experience of the RDCRN and the data presented in this study demonstrate that direct patient engagement in the development of research networks and design and conduct of research projects is feasible, and highly regarded in the conduct of clinical research in rare diseases. PAGs are ready, willing, and able to participate in all aspects of research, including study design, recruitment, research prioritization, funding strategies, and dissemination of research results. The support and direction provided by the Office of Rare Diseases Research within the NCATS for the RDCRN was also essential for the development of the investigator-patient partnerships. The experience of the RDCRN can be applied to other groups studying rare diseases and to groups studying more common disorders. RDCRN principal investigators and leaders of the associated PAGs all agree that the quality, quantity, efficiency, and pace of clinical research in rare diseases is greatly enhanced by the types of investigator-patient partnerships that have developed within the RDCRN.

These results are consistent with other studies of patient engagement [9–12]. Those studies also identified the key role that PAGs play in patient recruitment identified by academic researchers and PAGs alike, reflecting the highly positive results of PAG interactions with investigators. This study adds to that literature by highlighting the area of communication and dissemination of study results. However, these results differ from the CTTI report [9] that also found patient group respondents valued their contributions to research protocol development, funding, and interpretation of study results more highly than those contributions were valued by academic respondents. This RDCRN study found that these components of interaction were generally equally rated in terms of impact and importance by PAGs and Consortium PIs. The reluctance to share information perceived by investigators and patient group members in the CTTI study was not found in this RDCRN study in which PAGs were seen as playing an important role in dissemination of information and study results. This may be due to the way that PAGs have been integrated from the start into RDCRN Consortium activities and are consulted in study design and development. Through monthly RDCRN-CPAG conference calls and annual in-person meetings, PAGs also have the opportunity to share their experiences, learn from each other, and gather information about resources and scientific programs at ORDR, NCATS, and other Institutes of NIH.

The RDCRN has utilized its strong platform for collaboration between PAGs and investigators to produce a highly productive synergy that can serve as a model of patient engagement in conducting clinical research, especially in rare diseases. Additionally, this development of substantial patient engagement has transformed the work of the RDCRN and is a model for collaborative research.

## Conclusions

Results suggest active involvement by PAGs contribute to shaping Consortias’ research agendas and contribute to ensuring the feasibility and success of research protocols by assisting with patient recruitment. This extensive involvement is the basis for the positive impact of PAG-Investigator partnership in the RDCRN.

## Ethics approval

The survey was reviewed and approved by the University of South Florida Institutional Review Board. The Institutional Review Board approved a waiver of the requirements for the documentation of informed consent.

## Consent for publication

Not Applicable.

## Availability of data and material

Consistent with the Rare Diseases Clinical Research Network’s data sharing policy, data sets will be available on the Rare Diseases Clinical Research Network Public Website (www.rdcrn.org).

## References

[1] Health Promotion and Disease Prevention Amendments of 1984, Pub. L. No. 98–551, 98 Stat. 2815 (Oct. 30, 1984).

[2] Groft SC, Gopal-Srivastava R (2013). A model for collaborative clinical research in rare diseases: experience from the Rare Disease Clinical Research Network program. J Clin Invest.

[3] Krischer JP, Gopal-Srivastava R, Groft SC, Eckstein DJ, Rare Diseases Clinical Research Network (2014). The Rare Diseases Clinical Research Network's organization and approach to observational research and health outcomes research. J Gen Intern Med.

[4] Akers A, Ball KL, Clancy M (2013). Brain Vascular Malformation Consortium: overview, progress, and future directions. J Rare Disord.

[5] Chronic Graft Versus Host Disease Consortium (2011). Rationale and design of the chronic GVHD cohort study: improving outcomes assessment in chronic GVHD. Biol Blood Marrow Transplant.

[6] Gadegbeku CA, Gipson DS, Holzman LB (2013). Design of the Nephrotic Syndrome Study Network (NEPTUNE) to evaluate primary glomerular nephropathy by a multidisciplinary approach. Kidney Int.

[7] Richesson RL, Lee HS, Cuthbertson D, Lloyd J, Young K, Krischer JP (2009). An automated communications system in a Contact Registry for persons with rare diseases: scalable tools for identifying and recruiting clinical research participants. Contemp Clin Trials.

[8] Richesson RL, Sutphen R, Shereff D, Krischer JP (2012). The Rare Diseases Clinical Research Network Contact Registry update: features and functionality. Contemp Clin Trials.

[9] Clinical Trials Transformation Initiative. CTTI website. http://www.ctti-clinicaltrials.org/. Accessed Feb 2016.

[10] Landy DC, Brinich MA, Colten ME, Horn EJ, Terry SF, Sharp RR (2012). How disease advocacy organizations participate in clinical research: a survey of genetic organizations. Genet Med.

[11] Gallin EK, Bond E, Califf RM, Crowley WF, Davis P, Galbraith R (2013). Forging stronger partnerships between academic health centers and patient-driven organizations. Acad Med.

[12] Forsythe LP, Szdlowski V, Murad MH, Ip S, Wang Z, Elraiyah TA, Fleurence R, Hickam DH. A systematic review of approaches for engaging patients for research on rare diseases. J Gen Intern Med. 29(Suppl 3):S788–800. doi:10.1007/s11606-014-2895-9. 10.1007/s11606-014-2895-9 PMC4124116 25047393

